# Advancing analytical methodologies for the determination of lifestyle and dietary biomarkers in municipal wastewater

**DOI:** 10.1007/s00216-026-06495-1

**Published:** 2026-04-30

**Authors:** Karlo Jambrosic, Tin Zupanovic, Ivona Krizman-Matasic, Senka Terzic, Marijan Ahel, Ivan Senta

**Affiliations:** Division for Marine and Environmental Research, Rudjer Boskovic Institute, Bijenicka 54, 10000 Zagreb, Croatia

**Keywords:** Food biomarkers, LC–MS/MS, Method development, Stability, Wastewater analysis, Wastewater-based epidemiology

## Abstract

Wastewater-based epidemiology (WBE) is increasingly used to assess the consumption of illicit drugs and other lifestyle chemicals, while its potential for evaluating population dietary habits has been scarcely investigated. In this study, a multi-residue analytical method was developed to determine more than 40 lifestyle and dietary biomarkers in raw municipal wastewater. In addition to biomarkers commonly included in WBE studies, this method incorporates several food biomarkers not previously analysed in this context. The target biomarkers were analysed using ultra-high-performance liquid chromatography–tandem mass spectrometry. Optimal separation was achieved on an ACQUITY UPLC HSS T3 column using gradient elution with 0.1% acetic acid and acetonitrile, while multiple reaction monitoring mode was used for detection and quantification of the target compounds. Sample preparation involved solid-phase extraction (SPE) on hydrophilic-lipophilic cartridges for biomarkers present at lower concentrations in wastewater (mainly food biomarkers), while direct injection of diluted wastewater samples was used for other analytes, including alcohol and tobacco/nicotine biomarkers, artificial sweeteners, antidiabetic metformin, certain vitamins, and a few other food biomarkers. The method validation parameters, including SPE recovery and accuracy, were acceptable in most cases, and the method sensitivity was sufficient to enable reliable determination of the target biomarkers in real samples. Regarding stability in wastewater during sample collection and storage, most biomarkers remained stable at 4 °C for 24 h, but moderate to high loss of some analytes occurred during the freeze–thaw cycle. The method’s applicability was verified using 24-h wastewater samples from four Croatian cities, confirming its suitability for WBE.

**Graphical abstract:**

**Figure:**
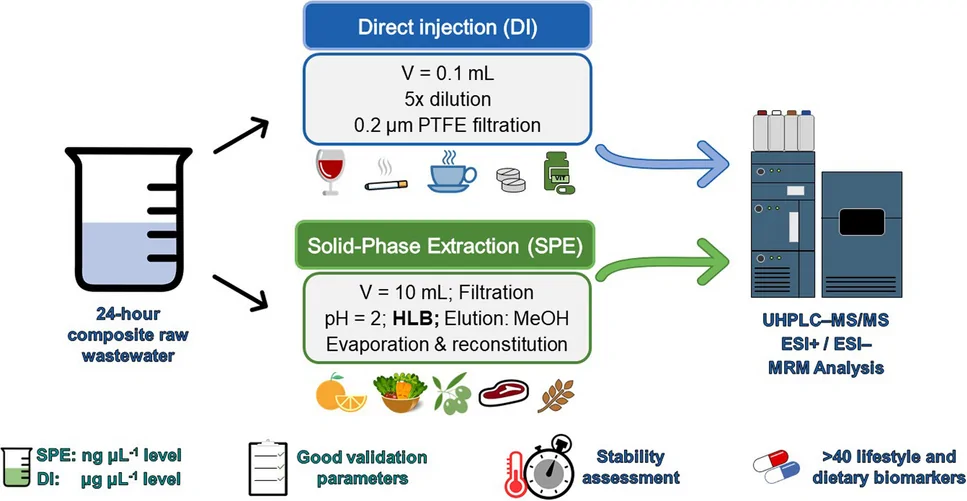

**Supplementary Information:**

The online version contains supplementary material available at https://doi.org/10.1007/s00216-026-06495-1.

## Introduction

Wastewater-based epidemiology (WBE) is a relatively new approach for obtaining some relevant epidemiological information at the population level. It relies on the chemical analysis of specific urinary biomarkers, excreted by the human body, in raw municipal wastewater [1]. WBE is now widely recognised as an objective, cost-effective, and timely approach for assessing illicit drug use at local [2], national [3], and international levels [4], and is particularly well suited to evaluating temporal trends in drug consumption [5]. In recent years, it has also been increasingly used to assess the consumption of other lifestyle chemicals such as alcohol [6], nicotine [7], caffeine [8], and artificial sweeteners [9].

In contrast, its potential for assessing the dietary habits of the population has hardly been investigated, as there are only a few published studies [10–14]. One reason for this may be the very large number of potential biomarkers, as more than 27,000 compounds have been described in food [15]. Most are not specific to a particular food type, and their metabolism is not always elucidated. In addition, two studies investigating the suitability of food biomarkers using laboratory-scale sewer reactors with biofilm or sediment have shown that most potential food biomarkers are significantly degraded under typical sewer conditions [16, 17]. Moreover, in many cases, their per capita load deviated from expected values, indicating additional sources (other than human metabolism) in wastewater. Nevertheless, these studies suggest that some food biomarkers could be useful for WBE, while two lignans (enterodiol and enterolactone) and proline betaine (a biomarker of citrus fruits) have been proposed as general indicators of healthy dietary habits and health outcomes [16]. In their pioneering work, Venkatesan et al. analysed three phytoestrogens (plant-derived human biomarkers), namely daidzein, genistein, and enterolactone, in wastewater to assess trends in plant-based diets in two US cities [11]. In a follow-up study, promising results were obtained with an integrated multi-omics approach combining WBE and genomic wastewater analysis [12], while Choi et al. used WBE to link lifestyle and dietary habits with social, demographic, and economic parameters of the population [10]. This study covered 22 wastewater treatment plants (WWTPs) and included 21% of the Australian population. It focused on 42 biomarkers from different classes, including some food biomarkers, mostly those previously shown to be promising for WBE, such as metabolites of vitamins B3, B6, and E, enterodiol, enterolactone, and proline betaine. The study demonstrated that WBE can be used to investigate socio-demographic influences and disparities in the chemical consumption footprint. In their study focusing on analytical aspects of biomarker screening in wastewater, Bernier-Turpin et al. also included some food biomarkers [13], mainly those proposed by Choi et al. However, most of these studies included only a very limited number of dietary biomarkers, given the enormous number of food constituents. Only two analytical methods have included a larger number of food biomarkers. The first is the method used in the two stability studies [16, 17]. However, this method could not detect several biomarkers (e.g. 3,5-dihydroxyphenylpropionoic acid, 3-carboxy-4-methyl-5-propyl-2-furanpropanoic acid, and two vitamin E catabolites), in real wastewater samples because it involves direct injection (without pre-concentration), resulting in lower sensitivity. Another multi-residue method, which includes several food biomarkers such as vitamins, lignans, isoflavones, amino acids, and gut metabolites, was published very recently [14]. However, this wide-scope analytical method included more than 200 biochemical indicators and therefore could not cover all aspects important to food biomarkers, such as their stability in wastewater.

The aim of this work was therefore to develop and validate a multi-residue analytical method for the determination of more than 40 lifestyle and dietary biomarkers in raw municipal wastewater, focusing on food biomarkers, particularly flavonoids and vitamins, but also including artificial sweeteners, the antidiabetic metformin, and biomarkers of alcohol, tobacco, and caffeine consumption. In addition to compounds already included in WBE studies, this method covers several dietary biomarkers that have not previously been analysed in this context. The study also assesses biomarker stability in wastewater during sample collection and storage, and demonstrates the method’s applicability using samples from four Croatian WWTPs. To the authors’ knowledge, this is one of the first multi-analytical methods suitable for WBE that allows quantification of a larger number of potential food biomarkers.

## Materials and methods

### Selection of biomarkers

The target biomarkers, their abbreviations, and primary sources are listed in Table 1, while their structures and basic properties are provided in Table S1.

**Table 1:** List of target biomarkers included in the study

Category	Biomarker of (main source)	Compound	Abbreviation
Alcoholic beverages	Total alcohol	Ethyl sulfate	EtS
	Wine	*cis*-Resveratrol	c-RES
		*trans*-Resveratrol	t-RES
	Beer	Isoxanthohumol	IXH
		8-Prenylnaringenin	PRE
Tobacco/nicotine products	Nicotine	Cotinine	COT
		Cotinine glucuronide	COT-Glu
		*trans*−3′-Hydroxycotinine	COT-OH
	Tobacco	Anabasine	ANB
Caffeine products	Caffeine	Caffeine	CAF
		Paraxanthine	PAR
Artificial sweeteners	Sucralose	Sucralose	SUC
	Acesulfame	Acesulfame	ACE
	Saccharin	Saccharin	SAC
	Cyclamate	Cyclamate	CYC
Plant-based food	Various fruit/vegetables	Enterodiol	EDL
		Enterolactone	ELC
	Citrus fruit	Proline betaine	PB
		Naringenin	NAR
		Hesperetin	HES
	Various vegetables/herbes	Apigenin	API
		Luteolin	LUT
	Cruciferous vegetables	Sulforaphane *N*-Acetyl cysteine	SNC
		*S*-Methyl-L-cysteine sulfoxide	MCS
	Soy and other legumes	Daidzein	DAI
		Equol	EQ
		Genistein	GEN
		*O*-desmethylangolensin	DMA
	Various plant-based food^1^	3,5-Dihydroxy-4-methoxybenzoic acid	DMBA
	Wholegrains	3,5-Dihydroxyphenylpropionoic acid	DHPPA
	Flaxseed	Secoisolariciresinol	SEC
	Olive oil	Tyrosol	TYR
		Hydroxytyrosol	TYR-OH
Animal-based food	Meat	Anserine	ANS
	Fish	3-Carboxy-4-methyl-5-propyl-2-furanpropanoic acid	CMPF
Vitamins	B2	Riboflavin	B2
		8-Hydroxymethylriboflavin	B2-OH
	B3	Nicotinamide	NAMD
	B5	Pantothenic acid	B5
	B6	4-Pyridoxic acid	4-PA
	E	2,5,7,8-Tetramethyl-2(2′-carboxyethyl)−6-hydroxychroman	α-CEHC
		2,7,8-Trimethyl-2-(beta-carboxyethyl)−6-hydroxychroman	γ-CEHC
Antidiabetic drugs	Metformin	Metformin	MET

^1^Red wine, tea, legumes, fruit, etc.

The biomarkers were selected to include the main representatives from each category, with particular emphasis on food biomarkers, which have been understudied in WBE to date. In addition to ethyl sulphate, a well-established biomarker of alcohol consumption [18], this study included potential biomarkers of frequently used alcoholic beverages: RES for wine, and IXH and PRE for beer. Although RES is present in various foods, a cohort study conducted in Spain showed that wine is the predominant source of RES (> 98%) in the diet [19], so it was proposed as a specific wine biomarker [20]. It should be noted that two stereoisomers of RES were included; in most foods, the amount of the *trans* isomer is higher, but it can convert to the *cis* isomer through exposure to UV/Vis radiation [21]. This study also included two potential beer biomarkers: IXH, proposed as a specific beer biomarker in traditional epidemiology, and its major metabolite PRE [22]. For nicotine, two major metabolites commonly used in WBE were included: COT and COT-OH. However, it has been shown that conjugated COT in wastewater significantly contributes to total COT loads [23, 24]. Therefore, to avoid cumbersome and time-consuming enzymatic deconjugation, which could affect method performance for other analytes, COT-Glu was also included in the method, while ANB was selected as a tobacco-specific biomarker as it could enable differentiation between tobacco and non-tobacco nicotine sources [25]. In addition to the parent compound, PAR was included as a specific CAF biomarker, as in most WBE studies [18], while ACE, CYC, SAC, and SUC were selected as biomarkers of common artificial sweeteners [9].

A large number of plant-based food biomarkers were selected, including those already used in WBE studies and several new potential biomarkers of fruit/vegetables, soy, flaxseed, and olive oil. These include the flavonoids NAR, HES, API, and LUT, as well as biomarkers from other chemical classes: MCS, DMA, DMBA, SEC, TYR, and TYR-OH. ANS and CMPF were selected as biomarkers of meat and fish consumption, respectively. In addition to the several vitamins already included in WBE studies, this method also included B2-OH, a metabolite of vitamin B2. Finally, the antidiabetic drug metformin was included. Besides its use in treating diabetes, metformin is also (ab)used for weight loss [26], indicating a potential link to dietary habits.

### Chemicals and materials

The CAS numbers and suppliers of reference materials, including analytes and isotopically labelled internal standards, are listed in Table S2. The analytical standards were obtained as pure materials (purity > 95%) or as solutions provided by the supplier. Individual stock solutions of analytes were prepared in LC–MS grade methanol at concentrations of 0.5–2 mg mL^−1^, except for α-CEHC, MCS, and B2, which were dissolved in DMSO, water, and 1% ammonium acetate in 50:50 (*v/v*) MeOH/H_2_O, respectively. Stock solutions were stored in amber glass at − 20 °C. Intermediate mixtures of all target analytes (1 ng mL^−1^ to 10 µg mL^−1^) were prepared in methanol. Isotopically labelled internal standards were prepared in the same way, with stock concentrations between 0.1 and 2 mg mL^−1^. A mixed working solution containing all isotopically labelled internal standards was prepared in methanol at 1 μg mL^−1^ and also stored at − 20 °C. The following solvents and reagents were used: methanol (VWR, HPLC grade; J.T. Baker, LC–MS grade), acetonitrile (J.T. Baker, LC–MS grade), water (VWR, LC–MS grade), formic acid (Honeywell Fluka, LC–MS grade), glacial acetic acid (Honeywell Fluka, LC–MS grade), phosphoric acid (Sigma-Aldrich, ACS grade), ammonium formate (Honeywell Fluka), ammonium acetate (Honeywell Fluka), DMSO (Kemika, p.a.), and ammonia in methanol, anhydrous, 7 N (Thermo Fisher Scientific). Ultrapure water used for sample preparation was produced with an Elix–Milli-Q system (Millipore). Filtration materials included GF/D glass fibre filters (2.7 µm; Whatman) and 0.2 µm polytetrafluoroethylene (PTFE) syringe filters (13 mm; Waters). In the preliminary experiments, several syringe filters with a pore size of 0.45 μm were tested: polyvinylidene difluoride, glass fibre, polypropylene, polyethersulphone, regenerated cellulose, PTFE, and water-wettable PTFE. Oasis hydrophilic-lipophilic balance (HLB), mixed-mode cation exchange (MCX) and mixed-mode anion exchange (MAX) 3 cc/60 mg cartridges (Waters) were used in the development of the solid-phase extraction (SPE) method.

### Sample collection and preparation

Raw wastewater samples for method development, validation, and model experiments were collected at the inlet of the WWTP of the city of Zagreb, Croatia, which serves a population of approximately 685,000. Additional samples, used to demonstrate the applicability of the method, were collected at the WWTPs of three smaller Croatian cities: Čakovec, Karlovac, and Bjelovar, serving populations of approximately 55,000, 45,000, and 30,000, respectively. The map showing the sampling locations is provided in Fig. S1. All samples were 24-h composites collected using refrigerated autosamplers operating at 4 °C. The samples were collected in pre-rinsed HDPE containers, transported to the laboratory on ice packs, and processed on the same day.

Sample preparation involved two separate procedures: direct injection and SPE. For direct injection, 0.1 mL of wastewater was diluted fivefold with LC–MS water, spiked with 5 ng of each internal standard, and filtered through 0.2 µm PTFE syringe filters. For SPE, 10 mL of wastewater was spiked with the internal standards (the same amount as for direct injection), filtered through GF/D filters and adjusted to pH 2 with H_3_PO₄. The samples were then loaded onto HLB cartridges, which had previously been conditioned with 3 mL of MeOH, 3 mL of ultrapure water and 3 mL of 25 mM H_3_PO₄ (pH 2). The cartridges were washed with 3 mL of ultrapure water, dried under nitrogen for 15 min, and eluted with 3 mL of MeOH. The eluates were evaporated to dryness at 40 °C under nitrogen using a TurboVap evaporator (Caliper Life Sciences), and reconstituted in 0.5 mL of LC–MS water to achieve a 20-fold concentration factor. All extracts were analysed on the same day, with each batch containing process and solvent blanks.

In the preliminary experiments, conducted in quadruplicate, spring water was used as a matrix to assess filtration losses on syringe filters and recovery on SPE cartridges. Filtration losses were determined using 0.5 mL of spring water spiked at 10 µg L^−1^, while SPE recovery was determined using 10 mL of spring water spiked at 2 µg L^−1^. HLB cartridges were tested at both pH 2 and without pH adjustment (pH 7–7.5). For MCX cartridges, samples were acidified to pH 2, while MAX cartridges were tested without pH adjustment. The preliminary SPE experiments with HLB cartridges also included selection of the elution solvent, and two solvents were tested: MeOH and 0.5% NH_3_ in MeOH. Meanwhile, 0.5% NH_3_ in MeOH and 2% HCOOH in MeOH were used to elute the MCX and MAX cartridges, respectively.

### Instrumental analysis

The target biomarkers were analysed using ultra-high-performance liquid chromatography–tandem mass spectrometry. The analyses were performed on an ACQUITY UPLC I-Class PLUS system coupled to a XEVO TQ-XS triple quadrupole mass spectrometer (Waters). Four ACQUITY UPLC chromatographic columns (Waters) were tested for the separation of the target compounds: HSS T3 (100 × 2.1 mm, 1.8 µm), BEH C18 (100 × 2.1 mm, 1.7 µm), CSH C18 (50 × 2.1 mm, 1.7 µm) and CSH Phenyl-Hexyl (50 × 2.1 mm, 1.7 µm). An HSS T3 column with an HSS T3 guard column (5 × 2.1 mm, 1.8 µm) at 25 °C was used in the final method. The flow rate was 0.4 mL min^−1^ and the injection volume was 5 µL. Several eluents were also tested, and finally water with 0.1% (*v/v*) acetic acid (A) and acetonitrile (B) were selected for both negative and positive ionisation polarity. The gradient in negative mode (expressed as % of eluent A) was as follows: 0–1 min, 99; 1–2 min, 80; 2–7 min, 50; 7–10 min, 5; 10–11.5 min, 5; 11.5–12 min, 99; 12–13.5 min, 99. The gradient in positive mode was: 0–1 min, 99; 1–2 min, 80; 2–4 min, 70; 4–4.5 min, 5; 4.5–6 min, 5; 6–6.5 min, 99; 6.5–8 min, 99. Electrospray ionisation (ESI) was used in both polarities. The capillary voltage was 2.5 kV in negative mode and 0.5 kV in positive mode. The source temperature was 120 °C, and the desolvation temperature was 500 °C. The desolvation gas flow was 1000 L h^−1^, and the cone gas flow was 150 L h^−1^. Argon was used as the collision gas at 0.15–0.17 mL min^−1^. The acquisition was performed in multiple reaction monitoring (MRM) mode. Two characteristic transitions were selected for each target biomarker. In ESI(+), the precursors were [M+H]⁺, except for sucralose ([M+Na]⁺), while in ESI(−) the precursors were [M−H]^−^. The first transition was used for quantification, and the second, together with the ion ratio, was used for confirmation. Collision energies were optimised individually for each analyte by infusing standard solutions at 10 ng mL^−1^. The transitions, cone voltages, and collision energies are listed in Table 2. Scheduled MRM was applied with retention time windows of 0.6 min centred on the expected retention times. The dwell time was 8–20 ms per transition, depending on the number of concurrent transitions.

**Table 2:** Chromatographic and mass spectrometric parameters used for the MRM acquisition mode

Compound abbreviation	Internal standard	Polarity	Retention time (min)	Retention factor (*k*)^3^	Parent ion (*m*/*z*)	CV (V)	Product ion 1 (*m*/*z*)	CE 1 (V)	Product ion 2 (*m*/*z*)	CE 2 (V)
EtS	EtS-*d*_5_	-	1.43	1.2	124.9	34	96.8	22	79.8	14
c-RES	API-*d*_5_	-	5.71	7.9	227.1	20	185.0	18	143.0	24
t-RES	t-RES-^13^C_6_	-	5.10	7.0	227.1	20	185.0	18	143.0	24
IXH	EQ-*d*_4_	-	7.17	10.2	353.1	20	232.9	18	119.0	28
PRE	API-*d*_5_	-	8.37	12.1	339.0	50	219.0	20	132.9	28
COT	COT-*d*_3_	+	1.84	1.9	117.1	20	80.1	20	98.1	18
COT-Glu	COT-Glu-*d*_3_	+	1.09	0.7	353.2	2	79.9	48	118.0	46
COT-OH	COT-OH-*d*_3_	+	1.66	1.6	193.1	48	79.9	22	134.2	16
ANB	ANB-*d*_4_	+	1.75	1.7	163.1	16	94.0	14	117.8	20
CAF	CAF-^13^C_3_	+	2.85	3.5	195.1	14	138.0	18	110.0	24
PAR	CAF-^13^C_3_	+	2.46	2.8	181.0	24	68.9	26	123.9	18
SUC	SUC-*d*_6_	+	3.25	4.1	419.0	22	220.9	20	238.9	24
ACE	ACE-*d*_4_	-	2.80	3.4	162.1	10	81.8	12	77.7	22
SAC	SAC-*d*_4_	-	3.05	3.8	181.9	54	105.8	18	41.9	20
CYC	CYC-*d*_11_	-	3.15	3.9	178.0	6	79.8	24	95.7	20
EDL	EDL-^13^C_3_	-	5.21	7.1	301.1	20	253.0	20	106.0	35
ELC	ELC-^13^C_3_	-	6.53	9.2	297.1	20	189.0	21	107.0	26
PB	COT-Glu-*d*_3_	+	0.83	0.3	144.0	10	58.0	20	84.0	20
NAR	EQ-*d*_4_	-	6.27	8.8	271.1	20	150.9	18	119.0	26
HES	GEN-*d*_4_	-	6.51	9.2	301.1	20	163.9	26	135.9	31
API	API-*d*_5_	-	6.25	8.8	269.0	62	116.9	30	151.0	22
LUT	GEN-*d*_4_	-	5.49	7.6	285.2	14	132.9	34	150.9	26
SNC	–^1^	+	2.78	3.3	341.1	22	71.9	48	113.9	26
MCS	MCS-*d*_3_	+	0.67	0.0	152.0	10	70.0	18	88.0	10
DAI	DAI-*d*_6_	-	5.23	7.2	253.1	20	91.0	36	133.0	33
EQ	EQ-*d*_4_	-	6.36	8.9	241.1	20	119.0	24	135.0	15
GEN	GEN-*d*_4_	-	6.32	8.9	269.1	20	132.9	30	159.0	30
DMA	EQ-*d*_4_	-	6.89	9.8	257.1	20	136.0	25	108.0	32
DMBA	ACE-*d*_4_	-	2.99	3.7	183.1	12	167.9	12	123.9	18
DHPPA	CYC-*d*_11_	-	3.07	3.8	181.1	20	95.1	21	137.0	14
SEC	API-*d*_5_	-	4.61	6.2	361.2	20	165.0	26	120.9	31
TYR	TYR-*d*_4_	-	3.19	4.0	137.1	20	106.1	18	119.0	17
TYR-OH	TYR-OH-*d*_4_	-	2.87	3.5	153.1	20	123.0	17	95.1	22
ANS	MCS-*d*_3_	+	0.62	0.0	241.0	14	108.9	22	167.0	16
CMPF	CMPF-*d*_3_	-	6.74	9.5	239.1	20	151.1	18	195.0	15
B2	B2-^13^C_4_, ^15^N_2_	+	2.96	3.6	377.0	40	243.0	24	172.0	38
B2-OH	B2-^13^C_4_, ^15^N_2_	+	2.45	2.8	393.1	10	170.0	44	259.0	22
NAMD	4-PY-*d*_3_^2^	+	1.66	1.6	122.9	32	53.0	25	80.0	18
B5	CYC-*d*_11_	-	2.69	3.2	218.3	58	88.2	12	146.4	12
4-PA	4-PA-*d*_3_	+	1.88	1.9	184.0	10	147.9	30	65.0	20
α-CEHC	γ-CEHC-*d*_5_	-	7.14	10.2	277	40	150.1	15	233.0	16
γ-CEHC	γ-CEHC-*d*_5_	-	6.52	9.2	263.0	16	149.1	22	219.0	14
MET	MET-*d*_6_	+	0.96	0.5	130.1	4	60.0	12	71.0	20
EtS-*d*_5_	–	-	1.42	1.2	130.0	12	97.8	14	–	–
t-RES-^13^C_6_	–	-	5.09	7.0	233.2	12	191.0	18	–	–
COT-*d*_3_	–	+	1.83	1.9	180.1	20	80.1	20	–	–
COT-Glu-*d*_3_	–	+	1.05	0.6	356.2	2	79.9	42	–	–
COT-OH-*d*_3_	–	+	1.66	1.6	196.1	20	80.1	25	–	–
ANB-*d*_4_	–	+	1.74	1.7	167.1	26	121.7	20	–	–
CAF-^13^C_3_	–	+	2.85	3.5	198.0	20	140.0	22	–	–
SUC-*d*_6_	–	+	3.24	4.1	425.0	26	222.9	22	–	–
ACE-*d*_4_	–	-	2.79	3.4	166.1	14	85.8	14	–	–
SAC-*d*_4_	–	-	3.04	3.8	185.9	48	105.8	18	–	–
CYC-*d*_11_	–	-	3.14	3.9	189.1	56	79.9	24	–	–
EDL-^13^C_3_	–	-	5.21	7.1	304.3	12	255.2	24	–	–
ELC-^13^C_3_	–	-	6.53	9.2	300.2	10	121.9	22	–	–
API-*d*_5_	–	-	6.23	8.7	274.3	16	119.6	36	–	–
MCS-*d*_3_	–	+	0.66	0.0	155.1	10	88.0	10	–	–
DAI-*d*_6_	–	-	5.22	7.2	259.2	14	92.8	36	–	–
EQ-*d*_4_	–	-	6.35	8.9	245.2	12	122.9	12	–	–
GEN-*d*_4_	–	-	6.32	8.9	273.2	20	136.8	34	–	–
TYR-*d*_4_	–	-	3.18	4.0	141.1	10	107.9	16	–	–
TYR-OH-*d*_4_	–	-	2.86	3.5	157.2	12	124.9	14	–	–
CMPF-*d*_3_	–	-	6.73	9.5	242.2	10	154.0	16	–	–
B2-^13^C_4_, ^15^N_2_	–	+	2.96	3.6	383.1	10	249.0	22	–	–
4-PY-*d*_3_	–	+	1.80	1.8	156.0	10	95.1	22	–	–
4-PA-*d*_3_	–	+	1.87	1.9	187.0	20	149.9	20	–	–
γ-CEHC-*d*_5_	–	-	6.52	9.2	268.1	4	149.2	22	–	–
MET-*d*_6_	–	+	0.97	0.5	136.1	2	60.0	12	–	–

^1^External calibration

^2^*N*-methyl-4-pyridone-5-carboxamide-*d*_3_

^3^Calculation based on column specifications

*CV*, cone voltage; *CE*, collision energy

### Quantification and method validation

Data processing and quantification were performed in MassLynx 4.2 with TargetLynx XS V4.2 (Waters). Calibration standards were prepared by diluting intermediate mixtures with ultrapure water. Ten calibration points covered the concentration range from 50 pg mL^−1^ to 50 ng mL^−1^, with internal standards at a concentration of 10 ng mL^−1^. Linear regression with 1/x weighting was applied. To control carryover, a solvent blank was injected after the highest standard, and quality controls were run regularly during each batch and at the end of the sequence. Where available, isotopically labelled analogues were used as internal standards for quantification (Table 2). For the remaining analytes, the most appropriate internal standard was selected based on structural similarity and/or behaviour in wastewater (e.g. similar matrix effect) to achieve the highest possible accuracy. As no suitable internal standard was identified for SNC, external calibration was used for quantification. Compound identification was based on agreement of retention times and the ratio of quantification to confirmation transitions (± 20%).

The instrumental detection limits (IDLs) were determined using standards prepared in ultrapure water, defined as the lowest concentration giving a signal-to-noise ratio (*S*/*N*) of 3. The method quantification limits (MQLs) were extrapolated from wastewater samples as the lowest value corresponding to *S*/*N* ≈ 10 in the matrix, accounting for sample preparation (fivefold dilution for direct injection and 20-fold concentration for SPE).

The accuracy (trueness and precision), matrix effect, extraction recovery (for the SPE method), and filtration loss (for the direct injection method) were determined in model experiments performed in quadruplicate with real wastewater. Trueness and precision were assessed at three levels per method (direct injection: 5, 20, 50 µg L^−1^; SPE: 0.2, 0.5, 2 µg L^−1^), while the other validation parameters were determined at one level (direct injection: 20 µg L^−1^; SPE: 2 µg L^−1^). Trueness was calculated as follows:

1 $$\mathrm{Trueness} \left(\%\right)=\left(\frac{{c}_{2}-{c}_{1}}{{c}_{0}}\right)\times 100$$

where *c*_0_ is the nominal spiked concentration, *c*_1_ is the average concentration measured in the original (non-spiked) sample, and *c*_2_ is the average concentration measured in the spiked sample. Precision (repeatability) was expressed as the RSD (%) of the spiked samples.

The matrix effect (ME) was determined by comparing the mean peak area of the analytes spiked into the final extracts (*A*_*fe*_) with the mean response in the corresponding standard of the same concentration (*A*_*std*_), while correcting for analyte already present in the original sample (*A*_*orig*_):

2 $$Matrix\;effect \;\left(\%\right)=\frac{\left({A}_{fe}-{A}_{orig}-{A}_{std}\right)}{{A}_{std}}\times 100$$

Negative matrix effect values therefore indicate signal suppression, while positive values indicate signal enhancement.

The extraction recovery for the SPE method was determined using samples spiked before and after extraction, considering analytes already present in the original sample:

3 $$Extraction\;recovery \left(\%\right)=\frac{\left({A}_{be}-{A}_{orig}\right)}{{(A}_{ae}- {A}_{orig})}\times 100$$

where *A*_*be*_ is the average response in the sample spiked before extraction, *A*_*ae*_ is the average response in the sample spiked after extraction, and *A*_*orig*_ is the average response in the non-spiked extract.

The filtration loss for the direct injection method was determined from the previously filtered wastewater sample spiked with the target compounds using the following equation:

4 $$Filtration\;loss \left(\%\right)=\left(1- \frac{{A}_{f}}{{A}_{nf}}\right) \times 100$$

where *A*_*nf*_ is the average response in the unfiltered sample, and *A*_*f*_ is the average response in the filtered sample.

### Analyte stability

The 24-h in-sample stability was assessed in a model experiment using raw, non-sterilised wastewater to simulate the collection of 24-h composite samples [27]. The experiment was performed in triplicate at two temperatures. A 250 mL wastewater sample was spiked at 50 µg L^−1^ and divided into six Erlenmeyer flasks, which were stored at 4 °C and 25 °C in the dark. Aliquots were collected at 0, 2, 4, 6, 8, and 24 h, diluted with ultrapure water, and spiked with internal standards. After filtration through 0.2 µm PTFE syringe filters, the aliquots were analysed using the developed instrumental method, and stability was calculated using the following equation:

5 $$Stability \left(\%\right)= \left(\frac{{A}_{t}}{{A}_{0}}\right)\times 100$$

where ${A}_{t}$ is the average response at a given time point and ${A}_{0}$ is the average response at *t* = 0 h.

The observed changes reflect overall in-sample behaviour in raw wastewater and may include both abiotic processes and biologically mediated transformations. The second model experiment was conducted to assess the stability of target biomarkers at −18 °C, i.e. during the freeze–thaw cycle, which is relevant if samples are not processed and analysed immediately after collection. This experiment was also performed in triplicate using wastewater sample spiked at 1 µg L^−1^ for the SPE method and 10 µg L^−1^ for the direct injection method. One set of aliquots was processed and analysed immediately, while the other was processed and analysed after 30 and 95 days using the methods described previously.

## Results and discussion

### LC–MS/MS method optimisation

In the initial phase of method development, three MRM transitions were selected for each analyte, and two transitions for the internal standards. After injecting analytical standards and real wastewater samples, two transitions were retained for the analytes and one transition for the internal standards to reduce the total number of MRM transitions (Table 2). The selection was based on both signal intensity and specificity, i.e. potential interferences from the wastewater matrix. All parent ions were [M−H]^−^ or [M+H]⁺, except for sucralose, for which the sodium adduct, [M+Na]⁺, was selected due to its higher intensity, as in some previous studies [28]. Sucralose was also tested in negative polarity as [M−H]^−^, but the signal was lower and more variable than in positive polarity. Some authors have recommended using acetate [29] or chloride adducts [30] in negative mode; however, this option was not explored in this study. As expected, signal intensity was generally lower in negative ionisation polarity, particularly for highly polar, low-molecular-weight acidic compounds (e.g. EtS, SAC, CYC, TYR). However, this was not considered a major issue, as the concentrations of these compounds in wastewater are generally high enough to ensure their reliable determination.

Four chromatographic columns were tested for the separation of the target compounds, and HSS T3 was ultimately selected. Compared to BEH C18, the T3 phase increased retention and improved peak shapes of the most polar biomarkers (ANS, MCS, PB, MET, EtS, COT-OH) while maintaining narrow, symmetrical peaks. Phenyl-type phases (CSH Phenyl-Hexyl, CSH Fluoro-Phenyl) provided good separations for flavonoids, but performed poorly for highly polar compounds. Even with the T3 column, retention of the most polar analytes remained relatively low. The column dead time was estimated at 0.64 min. Accordingly, the earliest eluting analytes in positive mode (ANS, MCS, PB, and MET) exhibited very low retention factors (Table 2), with ANS and MCS eluting within the dead time. Although not optimal, this was considered acceptable in the context of the present multiresidue method. Hydrophilic interaction liquid chromatography (HILIC) would probably be more suitable for these highly polar compounds, but would require additional runs. Therefore, T3 was chosen as a compromise, starting the gradient with a high percentage (99%) of the aqueous mobile phase to maximise retention of the polar analytes. Regarding the composition of the mobile phase, several aqueous eluents were tested. The peak shapes of several analytes (e.g. ANB, SUC, LUT, MCS, DMBA, CMPF, B5, 4-PA) were unsatisfactory when pure water was used, as the chromatograms showed pronounced fronting, splitting, or baseline noise. Therefore, various additives, including formic acid, acetic acid, ammonium formate, and ammonium acetate, were evaluated. Formic and acetic acid generally sharpened the peaks, but reduced signal intensity in ESI(−), with this effect particularly pronounced for formic acid. The addition of ammonium buffers did not result in significant improvement, although the peak shape of some analytes, such as SNC, improved with ammonium acetate. Some methods suggest using ammonium fluoride as an eluent modifier in negative polarity [14]; however, this option was not examined in this study. Consequently, 0.1% acetic acid and acetonitrile were selected as eluents for both polarities. Using the same column and eluents in both polarities was very convenient for daily work. Although the same HSS T3 column and mobile phases were used in both ionisation modes, compounds determined in ESI(+) and ESI(−) were analysed in separate runs to obtain an adequate number of data points across chromatographic peaks, while maintaining sufficient dwell time to ensure robust and sensitive MRM acquisition. The gradients were optimised separately for ESI(+) and ESI(−) due to differences in retention behaviour and chromatographic separation requirements. For compounds analysed in positive mode, all target analytes eluted satisfactorily within a shorter gradient and a total run time of 8 min, whereas a longer run time of 13.5 min was required for the separation of the negative-mode analytes. The representative chromatograms are shown in Fig. S2. It can be seen that full separation of some target compounds was not achieved; however, this was not considered a major issue, as specific MRM transitions were used for all compounds except for the two isomers of resveratrol, which shared both transitions. However, their reliable determination was ensured by complete chromatographic separation. It should be noted that samples were injected from 100% water to minimise early-peak distortion.

### Optimisation of sample preparation

As this method includes more than 40 compounds from several classes, with different physico-chemical properties and concentrations in wastewater, the sample preparation was carefully optimised to enable their reliable and accurate determination. Due to the wide range of analyte concentrations in real samples, from low ng L^−1^ to several tens of μg L^−1^, two sample preparation techniques were used in the final method: direct injection of diluted samples and SPE. It should be noted that some additional compounds were initially included in the method development (e.g. *trans*-resveratrol-3-sulphate, isohumulone, quercetin, isorhamnetin, kaempferol, sulphoraphane cysteine, 1-methylhistidine, folic acid), but were eventually excluded because the method’s performance for these substances was not satisfactory.

#### SPE method

The SPE performance was initially assessed using three types of sorbents. HLB is a versatile polymeric reversed-phase SPE sorbent that can retain a wide range of compounds with different physico-chemical properties, while MAX and MCX are mixed-mode ion-exchange sorbents that can improve method selectivity for acidic and basic compounds, respectively. Sample pH and elution solvent were further optimised for HLB cartridges, whereas for ion-exchange sorbents, these parameters were pre-selected, considering the characteristics of each SPE sorbent and the acid–base properties of the target biomarkers.

**Fig. 1:**
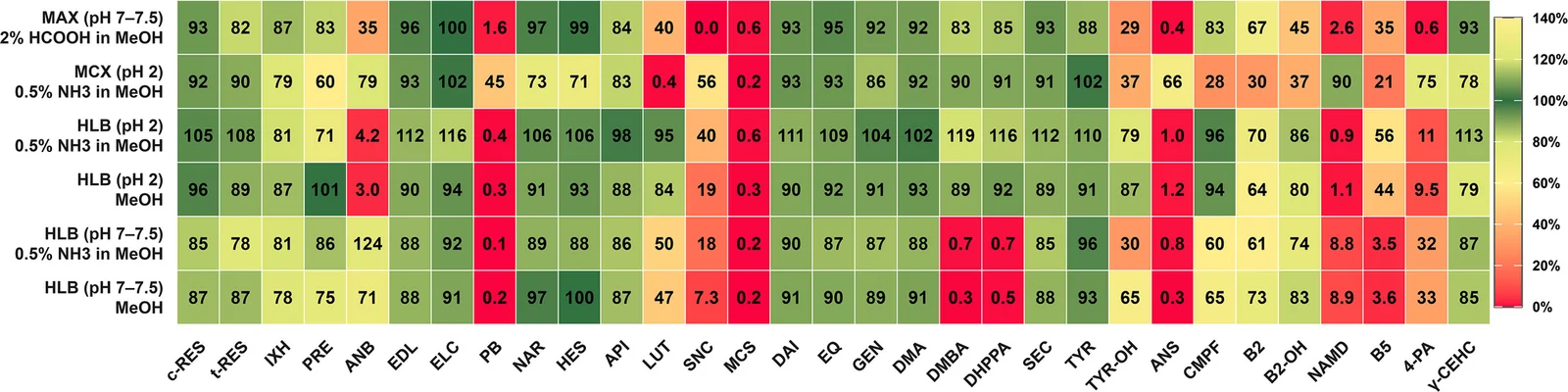
SPE recovery of the target compounds in spring water using the applied protocols (spiking level 2 μg L^−1^; *n* = 4). The colour gradient indicates the recovery value, ranging from low (red) to high (green), as shown on the colour scale to the right. The first line indicates the type of SPE cartridge used and the sample pH, while the second line lists the elution solvent

The recoveries of some target biomarkers were high (> 80%) with all the SPE sorbents tested (Fig. 1). These include most flavonoids and their metabolites, lignans, TYR, and γ-CEHC. However, this was not the case for the remaining analytes that showed low recovery with some or even all the SPE procedures tested. For example, recovery of ANS, NAMD, and 4-PA was acceptable only with MCX cartridges. However, the MCX sorbent did not perform well for some other biomarkers, such as LUT, CMPF, B2, and B2-OH, while the recovery of highly polar compounds, particularly PB and MCS, was low with all cartridges tested.

Sample pH was found to be an important parameter for the extraction of several compounds on HLB cartridges. For example, the recovery of DHPPA and DMBA was very low at the original pH (7–7.5), which can be explained by their acidic properties, as these compounds are present in anionic form at pH around 7. Their recovery was much higher at pH 2. The opposite situation was observed for weakly basic ANB. On the other hand, the influence of elution solvent (MeOH vs. 0.5% NH_3_ in MeOH) was less pronounced in most cases. The highest number of compounds could be efficiently extracted using the SPE protocol that includes percolation of acidified samples (pH 2) on HLB cartridges and elution with methanol, so this protocol was selected and used in the final method.

#### Direct injection method

Literature data and preliminary analysis of real wastewater samples indicated that the concentrations of several target biomarkers were sufficiently high (i.e. in the μg L^−1^ range) to allow their determination without a pre-concentration step, that is by direct injection. These include ethyl sulphate, nicotine and caffeine metabolites, artificial sweeteners, most vitamin biomarkers, and metformin. If the SPE method was used for their analysis, the concentrations in the final extract would require an extended method range, which might even exceed the optimal linear range of the instrument. Direct injection was also used for very polar target compounds that could not be efficiently retained on SPE sorbents (as mentioned above), and for compounds whose accuracy improved with direct injection (e.g. DHPPA and γ-CEHC).

The optimisation of the direct injection method focused on filtration and dilution. Syringe filters made from various materials with a pore size of 0.45 µm were tested to minimise potential analyte loss during filtration. Their performance was similar for most target compounds, although some differences were observed for specific analytes (Fig. S3). PTFE filters were ultimately selected for the final method. To prevent possible clogging of the column or instrument, filters with a pore size of 0.2 μm were used in the final method, that is, during validation and analysis of real wastewater samples.

The dilution of wastewater samples was applied because direct injection of raw wastewater, a heavily loaded matrix with high concentrations of various compounds, can rapidly reduce MS source cleanliness and impair instrument performance. Dilution also reduces matrix effects, which can be significant if sample preparation does not include SPE or other clean-up methods. However, dilution decreases method sensitivity, which may be relevant for compounds present at lower concentrations or with higher MQLs in wastewater. Therefore, a fivefold dilution was chosen as a compromise for the direct injection method.

### Method validation

The validation parameters for the SPE and direct injection methods are listed in Tables 3 and 4, respectively. The coefficients of determination (*r*^2^) for all target compounds were ≥ 0.99, while the IDLs were in the fg range (2.9 to 353 fg on-column).

**Table 3:** Method validation parameters (solid-phase extraction)

Compound abbreviation	*r*^2^	IDL (fg)	MQL (ng L^−1^)	SPE recovery (%)	Matrix effect (%)	Accuracy^1^ (%)		
						0.2 μg L^−1^	0.5 μg L^−1^	2 μg L^−1^
c-RES	0.994	140	36	85	−39	85 ± 14	75 ± 19	80 ± 18
t-RES	0.998	309	52	71	−44	94 ± 3	91 ± 2	99 ± 3
IXH	0.994	22	1.4	113	−44	116 ± 8	70 ± 11	81 ± 7
PRE	0.999	2.9	0.43	118	−65	77 ± 9	72 ± 13	90 ± 6
EDL	0.999	13	2.1	91	−20	126 ± 0	83 ± 1	100 ± 2
ELC	0.993	91	13	95	−18	105 ± 0	130 ± 8	114 ± 1
NAR	0.996	56	6.7	97	−25	140 ± 2	101 ± 2	116 ± 2
HES	0.999	8.3	4.5	97	−29	105 ± 5	80 ± 6	85 ± 4
API	0.994	159	15	103	−49	102 ± 3	84 ± 3	105 ± 2
LUT	0.994	92	15	99	−36	99 ± 7	105 ± 4	128 ± 2
SNC	0.994	50	33	85	48	129 ± 8	131 ± 1	127 ± 3
DAI	0.994	52	6.3	97	−39	122 ± 2	93 ± 2	107 ± 1
EQ	0.987	97	11	95	−28	100 ± 4	128 ± 18	108 ± 9
GEN	0.998	100	14	98	−34	95 ± 2	87 ± 2	91 ± 3
DMA	0.999	20	1.8	101	−28	99 ± 2	94 ± 4	107 ± 4
DMBA	0.998	240	16	94	−42	101 ± 3	79 ± 3	98 ± 2
SEC	0.992	56	27	92	−39	76 ± 11	74 ± 18	93 ± 13
TYR	0.996	6.2	105	90	−45	–^2^	104 ± 5	91 ± 10
TYR-OH	0.995	55	23	91	−13	131 ± 1	106 ± 3	115 ± 2
CMPF	0.994	140	12	95	0	110 ± 0	90 ± 2	103 ± 2
B2-OH	0.997	8.1	4.1	86	−12	100 ± 3	98 ± 2	103 ± 2

*IDL*, instrumental detection limit; *MQL*, method quantification limit; *SPE*, solid-phase extraction

^1^Trueness + Precision

^2^Not determined due to the high concentration in the original sample

**Table 4:** Method validation parameters (direct injection)

Compound abbreviation	*r*^2^	IDL (fg)	MQL (μg L^−1^)	Filtration loss (%)	Matrix effect (%)	Accuracy^1^ (%)		
						5 μg L^−1^	20 μg L^−1^	50 μg L^−1^
EtS	0.998	10	0.09	4	−47	94 ± 3	101 ± 2	97 ± 1
COT	0.994	4.8	0.03	4	−7	92 ± 3	94 ± 1	93 ± 2
COT-Glu	0.998	9.4	0.04	5	−7	101 ± 5	100 ± 2	91 ± 2
COT-OH	0.995	5.4	0.03	5	−5	90 ± 4	98 ± 2	94 ± 2
ANB	0.995	21	0.51	9	84	89 ± 3	91 ± 2	85 ± 1
CAF	0.997	12	0.10	3	−12	79 ± 4	100 ± 3	92 ± 1
PAR	0.998	16	0.08	6	−11	94 ± 3	99 ± 2	97 ± 1
SUC	0.991	312	2.1	4	−14	96 ± 2	95 ± 2	96 ± 1
ACE	0.996	20	0.11	3	−10	86 ± 3	97 ± 3	92 ± 1
SAC	0.996	56	0.33	3	−11	91 ± 3	102 ± 3	89 ± 1
CYC	0.996	29	0.16	2	−11	91 ± 3	96 ± 2	95 ± 2
PB	0.994	20	0.23	9	8	90 ± 7	123 ± 4	111 ± 3
MCS	0.996	5.4	0.06	15	−63	91 ± 4	99 ± 3	95 ± 1
DHPPA	0.997	270	1.4	1	−6	86 ± 3	95 ± 6	97 ± 5
ANS	0.998	31	0.20	10	−64	–^2^	111 ± 15	81 ± 23
B2	0.998	15	0.11	23	−41	105 ± 3	102 ± 2	90 ± 1
NAMD	0.994	22	0.06	1	−3	90 ± 3	93 ± 1	90 ± 1
B5	0.998	225	0.44	1	−7	88 ± 6	96 ± 5	92 ± 3
4-PA	0.996	41	0.23	30	0	93 ± 4	99 ± 2	96 ± 2
α-CEHC	0.998	81	0.32	2	−13	89 ± 5	114 ± 6	91 ± 6
γ-CEHC	0.992	353	1.5	3	−4	94 ± 8	126 ± 8	95 ± 7
MET	0.993	156	0.09	7	21	87 ± 3	97 ± 4	93 ± 1

*IDL*, instrumental detection limit; *MQL*, method quantification limit

^1^Trueness + Precision

^2^Not determined due to the high concentration in the original sample

The MQLs for the SPE method were mostly in the low ng L^−1^ range (0.43–52 ng L^−1^), except for TYR, whose MQL was 105 ng L^−1^. Most MQLs were comparable to those obtained in a recent WBE analytical method [14], with only a few exceptions. For example, the MQL of CMPF was nearly an order of magnitude lower with our method, while the opposite was observed for SNC [14]. The MQLs obtained in this study were in most cases also lower than in two other SPE-based WBE methods that included some representatives of food biomarkers [12, 13].

The MQLs for the direct injection method were generally higher, as expected, since they refer to the direct injection of diluted wastewater samples. However, in most cases, they remained well below the typical concentrations in raw wastewater and were mostly below or around 0.5 μg L^−1^ (0.03–0.51 μg L^−1^), except for SUC, DHPPA, and γ-CEHC, whose MQLs were in the low μg L^−1^ range (1.4–2.1 μg L^−1^). These MQLs were mostly similar to those obtained using a direct injection method by Choi et al. [10], with only a few exceptions, the most notable being sucralose and cotinine, whose MQLs differed by an order of magnitude in this study (higher for sucralose and lower for cotinine). It should be noted, however, that the MQLs reported in Tables 3 and 4 are only indicative, as they may vary between samples due to differences in wastewater composition. Therefore, the target compounds were quantified in individual samples if their *S*/*N* was equal to or greater than 10.

SPE recoveries in raw wastewater were ≥ 85% for all target compounds except t-RES (71%) and were generally similar to those determined in the preliminary experiment with ultrapure water (Fig. 1). Notably, the recovery of SNC in raw wastewater (85%) was much higher than in ultrapure water (19%). The recoveries obtained in this study were mostly similar to or higher than those reported in previous WBE analytical methods that included some food biomarkers. For example, recoveries of DAI, EDL, ELC, and EQ were comparable to those obtained with the method by Elliss et al., but recoveries of CMPF and GEN were much higher with our method [14]. Our recoveries were also generally higher than those reported in the other two WBE methods; however, those studies reported recovery for the entire analytical procedure, not just for SPE [11, 13], making direct comparison difficult.

Filtration losses in raw wastewater were mostly ≤ 10%, except for MCS, B2, and PA, which had losses of 15–30%. However, these losses were compensated by adding their isotopically labelled analogues as internal standards before filtration. Notably, losses of the vitamin E biomarkers α-CEHC and γ-CEHC were negligible in raw wastewater, although some losses were observed in ultrapure water (28% and 14%, respectively).

Matrix effects were generally low for compounds analysed by direct injection, except for the most polar compounds, which showed stronger signal reduction (EtS, MCS, ANS, B2) or enhancement (ANB). The matrix effects were similar to those obtained in the direct injection method by Choi et al., except for CAF and COT-OH, whose matrix effects were significantly lower in our study. On the other hand, low to moderate signal reduction (12–65%) was observed for target compounds analysed using the SPE method, except for CMPF, which showed no matrix effect, and SNC, which exhibited signal enhancement in wastewater (48%) (Table 3). Bernier-Turpin et al. reported stronger matrix effects (signal reduction) for the food biomarkers included in their method [13].

The method trueness was acceptable for all compounds analysed by direct injection, as it was mostly within the range of 80–120%. Slightly lower trueness was observed for some compounds analysed by the SPE method, mainly due to high background concentrations and/or the use of non-ideal internal standards. However, it was still between 70 and 130% in almost all cases, which can be considered acceptable for raw wastewater. The accuracy for most compounds was similar to that reported in the method by Elliss et al. The method precision was very good for all compounds analysed by direct injection (1–9%), except for ANS (15–23%). The precision was generally slightly lower for compounds analysed by the SPE method, but remained below 20% (0–19%). For SNC, however, no suitable isotopically labelled internal standard was available; therefore, the results for this compound should be considered semi-quantitative.

### Analyte stability

#### In-sample stability

**Fig. 2:**
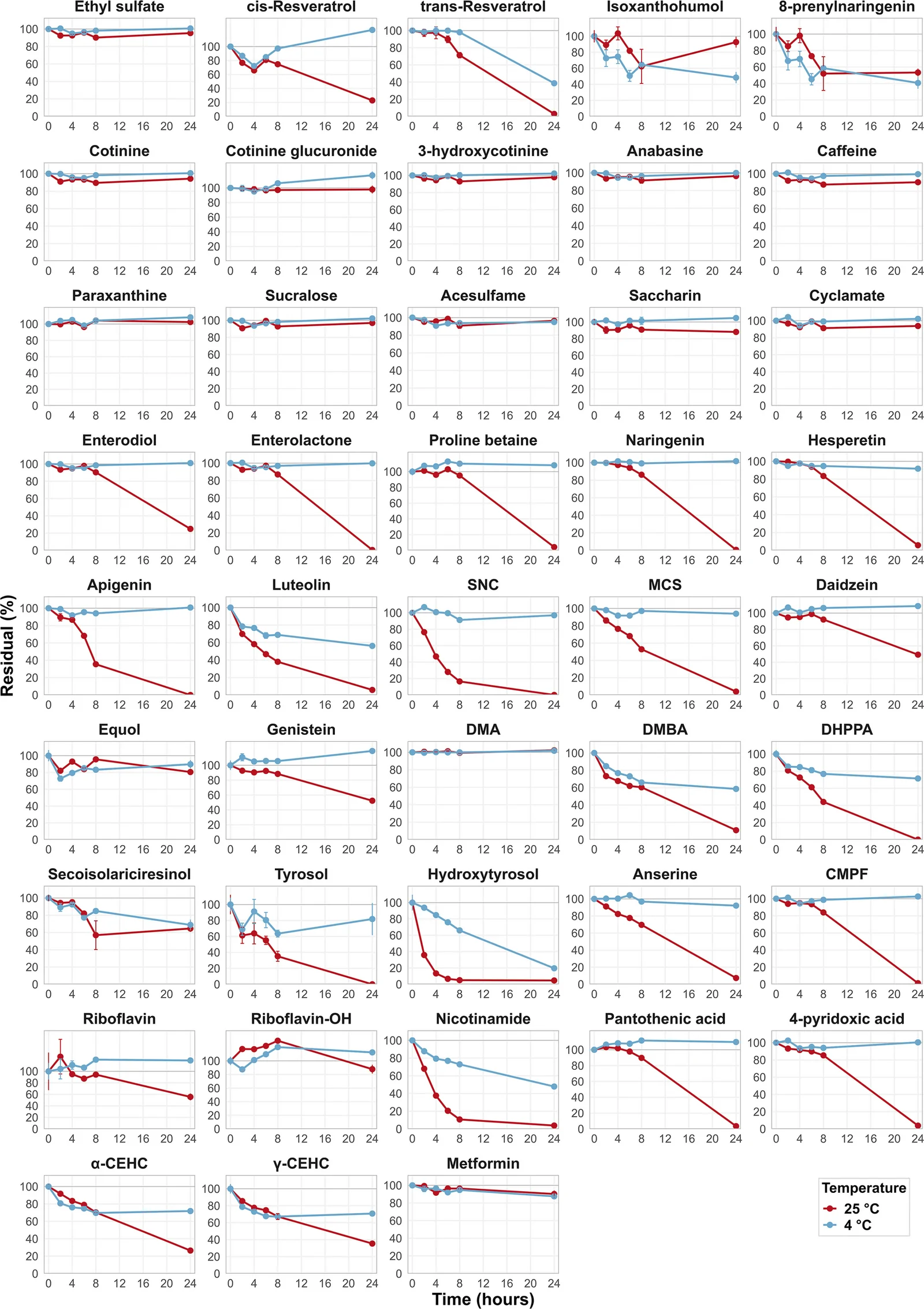
Twenty-four-hour stability of the target compounds in raw wastewater

Most target biomarkers remained stable at 4 °C in raw wastewater, with losses of less than 20% after 24 h (Fig. 2). Slightly higher losses, around 30%, were observed for DHPPA, SEC, α-CEHC, and γ-CEHC, while moderate losses, between 40 and 60%, were recorded for t-RES, IXH, PRE, LUT, DMBA, and NAMD. Only TYR-OH can be classified as very unstable, with a loss of 80% after 24 h.

Most lifestyle biomarkers, including EtS, MET, artificial sweeteners, and caffeine and tobacco biomarkers, were also stable at 25 °C. Their stability values were in fact very similar to those at 4 °C. However, this did not apply to most food biomarkers, whose stability at room temperature was significantly lower, with very few exceptions such as DMA. Interestingly, the loss of IXH was lower at 25 °C, which may be related to the re-transformation of conjugates, the main metabolic excretion products of IXH in humans [31], into the parent compound. Therefore, to obtain the most accurate figures for food biomarkers, sample collectors should always be refrigerated during the collection of 24-h composite wastewater samples. The same applies to the short-term storage of collected wastewater samples. Even then, the loss of several food biomarkers is not negligible.

It should be noted that these stability experiments were conducted solely to assess stability during 24-h composite sampling and short-term storage, while the possible influence of biofilm in the sewer pipes was not considered. It has been shown that the stability of WBE biomarkers is generally lower in the presence of a biofilm [16]. Therefore, additional experiments mimicking the conditions in real sewer systems should be conducted to further evaluate the suitability of the WBE biomarkers used in this study.

#### Freeze–thaw stability

Freezing is the standard procedure for sample preservation when immediate processing is not possible. However, some compounds may not remain stable during freeze–thaw cycles. Table S3 shows the stability of target biomarkers during storage at −18 °C for 30 and 95 days. While most compounds were stable for 30 days, with concentration changes below 20–25%, several analytes showed significant degradation. Moderate losses (30% to 60%) were observed for t-RES, NAR, HES, LUT, and SNC, while high losses (> 60%) were found for API, DMBA, TYR-OH, and NAMD. The stability of most biomarkers remained unchanged after freezing for 95 days. The main exceptions were the beer biomarkers IXH and PRE, which were stable for 30 days but showed high losses after 95 days. A similar pattern, with moderate losses after 95 days, was observed for GEN, O-DMA, DHPPA, TYR, B2, B2-OH, and γ-CEHC, while some biomarkers that already showed moderate degradation after 30 days, such as NAR, HES, and LUT, degraded further after 95 days. Therefore, for accurate quantitative determination of biomarkers with lower stability, samples should be processed as soon as possible after collection. If this is not feasible, using an appropriate preservation technique or adding internal standards before freezing may help. However, as this is not always possible, analyte losses should be taken into account.

### Application to real samples

**Fig. 3:**
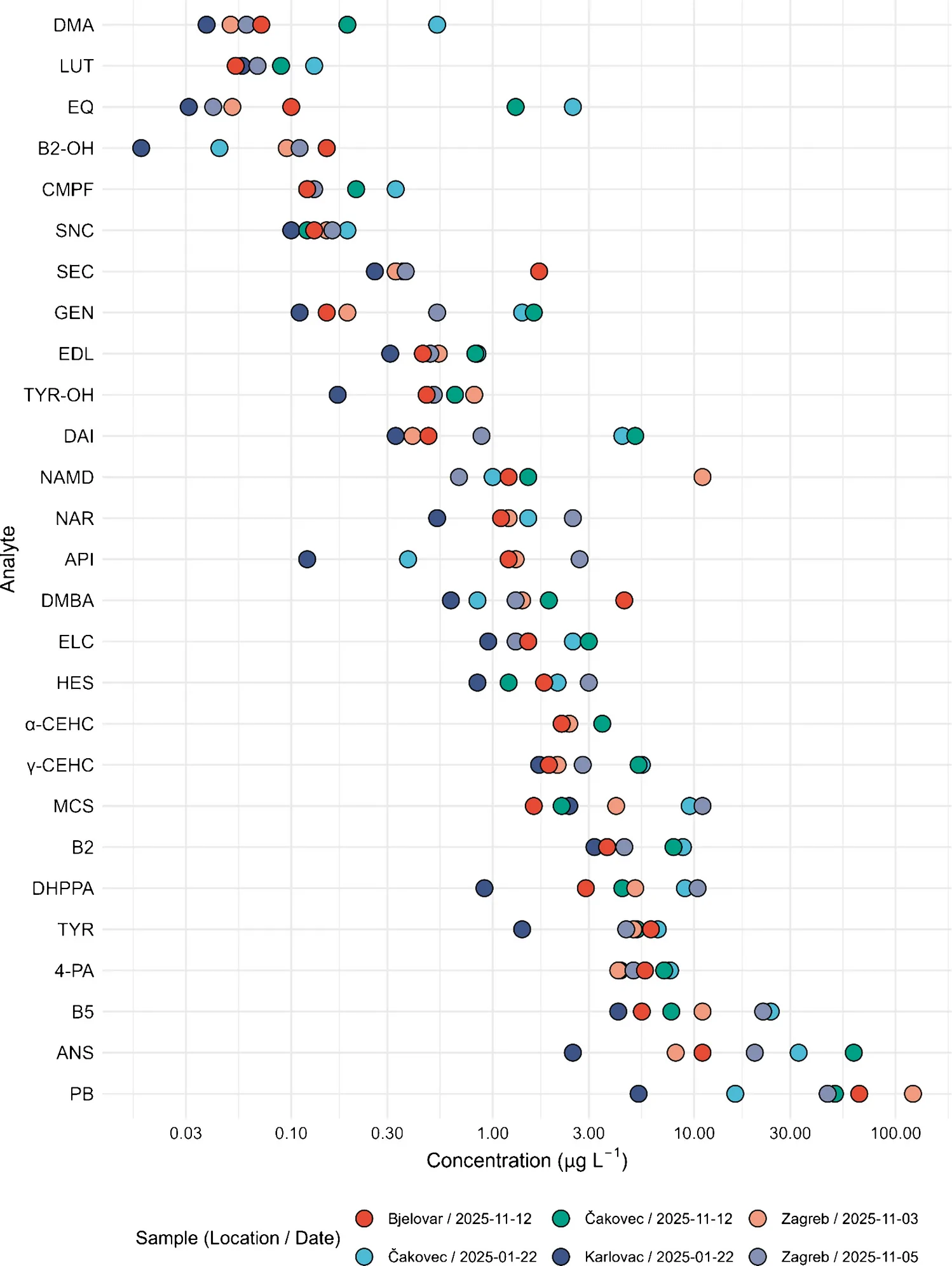
Concentrations of food biomarkers in raw wastewater samples from Croatian cities (log scale)

**Fig. 4:**
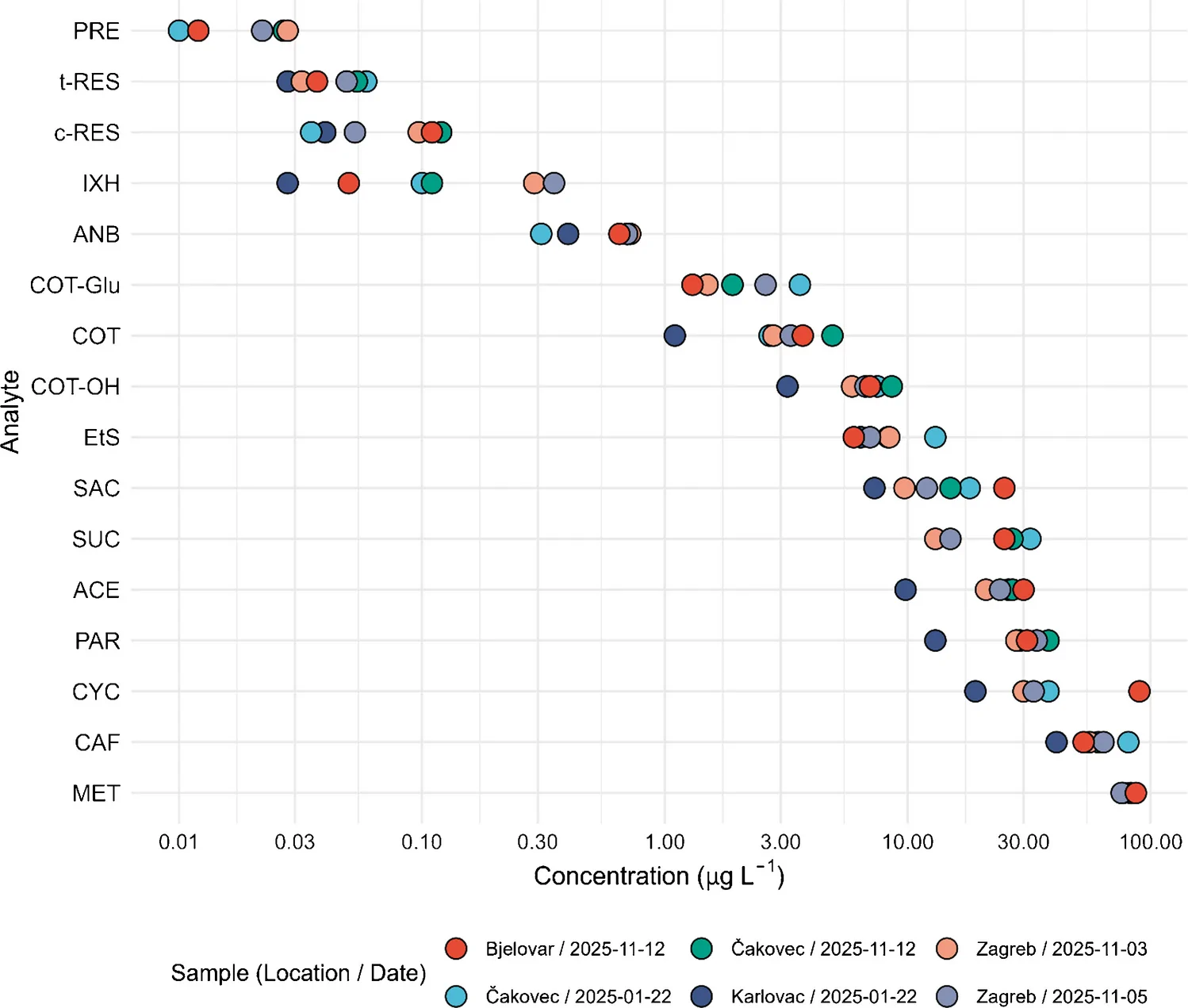
Concentrations of lifestyle biomarkers in raw wastewater samples from Croatian cities (log scale)

Target biomarkers were determined in all analysed wastewater samples using the developed analytical methods, with concentrations ranging from low ng L^−1^ to μg L^−1^ levels (Figs. 3, 4, and Table S4). These concentrations were generally consistent with those reported in previous WBE studies focusing on lifestyle and dietary biomarkers [6, 9–11, 13, 23, 32], except for ANB, whose concentrations were notably higher in our study compared to previous WBE studies [25, 33]. Another important observation concerns nicotine biomarkers. In the analysed wastewater samples, COT-Glu was present at concentrations comparable to those of COT (COT: 1.1–4.9 µg L^−1^; COT-Glu: 1.3–3.6 µg L^−1^; Table S4), indicating that the conjugated metabolite can contribute substantially to the overall cotinine mass loads in wastewater. This suggests that WBE studies relying solely on free cotinine may underestimate nicotine consumption. Therefore, nicotine-related WBE assessments should include either direct measurement of both COT and COT-Glu (as in this study) or an enzymatic deconjugation step before analysis [23]. A recent study demonstrated that the contribution of phase II metabolites can be significant not only for COT but also for some other biomarkers included in WBE studies [24].

The concentrations of most biomarkers were similar across all analysed samples. However, concentrations of soy biomarkers, particularly DAI, EQ, and GEN, were approximately an order of magnitude higher in both samples collected in Čakovec than in those from other cities. This may be related to the poultry industry, which is widespread in Čakovec, and suggests that some food biomarkers commonly used in WBE studies may not be suitable if effluents from the food industry contribute significantly. However, these initial observations should be confirmed in future studies with larger sample sizes to assess spatiotemporal trends and possible associations with demographic or socioeconomic factors.

## Conclusion

The developed analytical method enables accurate and reliable determination of over 40 lifestyle and dietary biomarkers in raw wastewater. It is suitable for WBE and includes several food biomarkers not previously analysed in this context. However, some of them are not sufficiently stable in wastewater, which should be considered when making quantitative assessments. The results also highlight the importance of including cotinine glucuronide, as omitting this conjugated metabolite may lead to underestimation of nicotine use. Further studies should verify the stability of selected biomarkers in real sewer systems and assess the potential contribution of phase II metabolites and non-human sources to determine their ultimate suitability for WBE.

## Supplementary Information

Below is the link to the electronic supplementary material.

Supplementary file1 (DOCX 2.83 MB)

## Data Availability

The datasets generated and analysed during the current study are available from the corresponding author on reasonable request.
