# Supplementary material for: Advancing analytical methodologies for the determination of lifestyle and dietary biomarkers in municipal wastewater
Source: Anal Bioanal Chem. 2026 Apr 30;418(18):6003–19. doi: 10.1007/s00216-026-06495-1 (PMC13541833; doi:10.1007/s00216-026-06495-1)
Supplement: Supplementary file 1 [file 216_2026_6495_MOESM1_ESM.docx]

**Advancing analytical methodologies for the determination of lifestyle and dietary biomarkers in municipal wastewater**

***SUPPLEMENTARY INFORMATION***

Karlo Jambrosic, Tin Zupanovic, Ivona Krizman-Matasic, Senka Terzic, Marijan Ahel, Ivan Senta*

Division for Marine and Environmental Research, Rudjer Boskovic Institute, Bijenicka 54, 10000 Zagreb, Croatia

* Corresponding author

E-mail: [isenta@irb.hr](mailto:isenta@irb.hr)

ORCID: 0000-0002-3308-5122

**Table S1.** Chemical classes, basic physico-chemical properties, and structures of the target compounds.

| **Compound** | **Chemical class / primary origin** | **Elemental composition** | **Molecular mass**  **(g mol^-1^)** | **log *K_ow_*** | ***p*K_a_** | **Chemical structure** |
| --- | --- | --- | --- | --- | --- | --- |
| Ethyl sulfate | Phase II ethanol metabolite  (sulphate ester) | C_2_H_6_O_4_S | 126.126 | -0.17 | -2.1 |  |
| *cis*-Resveratrol | Polyphenolic stilbenoid | C_14_H_12_O_3_ | 228.247 | 2.49 | 8.99  -5.7 |  |
| *trans*-Resveratrol | Polyphenolic stilbenoid | C_14_H_12_O_3_ | 228.247 | 2.48 | 8.49  -6.2 |  |
| Isoxanthohumol | Prenylated flavonoid (isoflavonoid) | C_21_H_22_O_5_ | 354.402 | 3.50 | 8.44  -3.3 |  |
| 8-Prenylnaringenin | Prenylated flavanone, phytoestrogen | C_20_H_20_O_5_ | 340.375 | 3.26 | 7.69  -5 |  |
| Cotinine | Nicotine metabolite (alkaloid) | C_10_H_12_N_2_O | 176.219 | 0.82 | 4.79* |  |
| Cotinine glucuronide | Phase II nicotine metabolite (*O*-glucuronide) | C_16_H_20_N_2_O_7_ | 352.343 | -2.75 | 2.85  -1.2 |  |
| *trans*-3'-Hydroxycotinine | Hydroxylated nicotine metabolite | C_10_H_12_N_2_O_2_ | 192.218 | 0 | 13.11  4.79 |  |
| Anabasine | Tobacco alkaloid | C_10_H_14_N_2_ | 162.236 | 1.52 | 9 |  |
| Caffeine | Methylxanthine stimulant | C_8_H_10_N_4_O_2_ | 194.194 | -0.08 | -0.92 |  |
| Paraxanthine | Dimethylxanthine (caffeine metabolite) | C_7_H_8_N_4_O_2_ | 180.167 | -0.07 | 10.76  -0.87 |  |
| Sucralose | Chlorinated disaccharide sweetener | C_12_H_19_Cl_3_O_8_ | 397.626 | -1.01 | 11.91  -3 |  |
| Acesulfame | Sulphonamide sweetener | C_4_H_4_NO_4_S | 163.147 | -0.23 | 3.02  -6 |  |
| Saccharin | Benzoic sulphonimide sweetener | C_7_H_5_NO_3_S | 183.181 | 0.60 | 1.94 |  |
| Cyclamate | Cyclohexylsulphamate sweetener | C_6_H_12_NSO_3_^–^ | 178.226 | 0.48 | -0.83  -9.7 |  |
| Enterodiol | Enterolignan (microbial lignan metabolite) | C_18_H_22_O_4_ | 302.370 | 2.46 | 9.79  -2.6 |  |
| Enterolactone | Enterolignan | C_18_H_18_O_4_ | 298.338 | 2.74 | 9.16  -6 |  |
| Proline betaine | Quaternary ammonium betaine | C_7_H_13_NO_2_ | 143.186 | -1.59 | 2.26 |  |
| Naringenin | Flavanone | C_15_H_12_O_5_ | 272.256 | 1.84 | 7.86  -5 |  |
| Hesperetin | Flavanone | C_16_H_14_O_6_ | 302.282 | 1.91 | 7.86  -4.6 |  |
| Apigenin | Flavone | C_15_H_10_O_5_ | 270.240 | 2.11 | 6.57  -5.4 |  |
| Luteolin | Flavone | C_15_H_10_O_6_ | 286.239 | 1.73 | 6.57  -5.4 |  |
| Sulforaphane *N*-Acetyl cysteine | Isothiocyanate conjugate (mercapturic acid) | C_11_H_20_N_2_O_4_S_3_ | 340.471 | 0.74 | 3.97  -2 |  |
| *S*-Methyl-L-cysteine sulfoxide | Sulphur-containing amino acid derivative | C_4_H_9_NO_3_S | 151.180 | -1.87 | 1.61  8.45 |  |
| Daidzein | Isoflavone phytoestrogen | C_15_H_10_O_4_ | 254.241 | 2.24 | 6.48  -5.3 |  |
| Equol | Isoflavandiol (microbial metabolite) | C_15_H_14_O_3_ | 242.274 | 2.57 | 9.63  -4.9 |  |
| Genistein | Isoflavone | C_15_H_10_O_5_ | 270.240 | 2.04 | 6.55  -5.3 |  |
| *O*-desmethylangolensin | Isoflavonoid metabolite | C_15_H_14_O_4_ | 258.273 | 2.28 | 7.83  -4.9 |  |
| 3,5-Dihydroxy-4-methoxybenzoic acid | Phenolic acid | C_8_H_8_O_5_ | 184.147 | 0.68 | 3.93  -4.9 |  |
| 3,5-Dihydroxyphenylpropionoic acid | Phenylpropionic acid (microbial metabolite) | C_9_H_10_O_4_ | 182.175 | 0.94 | 3.82  -5.7 |  |
| Secoisolariciresinol | Plant lignan | C_20_H_26_O_6_ | 362.422 | 2.46 | 9.96  -2.6 |  |
| Tyrosol | Phenolic alcohol | C_8_H_10_O_2_ | 138.166 | 1.10 | 10.2  -2.4 |  |
| Hydroxytyrosol | Phenolic alcohol | C_8_H_10_O_3_ | 154.165 | 0.56 | 9.45  -2.4 |  |
| Anserine | Histidine-containing dipeptide | C_10_H_16_N_4_O_3_ | 240.263 | -0.86 | 3.44  9.13 |  |
| 3-Carboxy-4-methyl-5-propyl-2-furanpropanoic acid | Urofuran acid (furan fatty acid metabolite) | C_12_H_16_O_5_ | 240.255 | 1.83 | 3.87  -2.9 |  |
| Riboflavin | Vitamin B2 (Riboflavin) | C_17_H_20_N_4_O_6_ | 376.369 | -0.19 | 5.97  -2.6 |  |
| 8-Hydroxymethylriboflavin | Riboflavin metabolite | C_17_H_20_N_4_O_7_ | 392.368 | -1.03 | 9.76** |  |
| Nicotinamide | Vitamin B3 amide | C_6_H_6_N_2_O | 122.127 | 0.64 | 13.39  3.63 |  |
| Pantothenic acid | Vitamin B5 | C_9_H_17_NO_5_ | 219.237 | -0.51 | 4.35  -2.8 |  |
| 4-Pyridoxic acid | Vitamin B6 catabolite | C_8_H_9_NO_4_ | 183.163 | -0.10 | 2.55  4.09 |  |
| 2,5,7,8-Tetramethyl-2(2′-carboxyethyl)-6-hydroxychroman | Vitamin E (α-tocopherol) metabolite | C_16_H_22_O_4_ | 278.348 | 2.98 | 4.4  -4.9 |  |
| 2,7,8-Trimethyl-2-(beta-carboxyethyl)-6-hydroxychroman | Vitamin E (γ-tocopherol) metabolite | C_15_H_20_O_4_ | 264.321 | 2.68 | 4.3  -4.9 |  |
| Metformin | Biguanide antidiabetic drug | C_4_H_11_N_5_ | 129.167 | -0.75 | 12.33 |  |

Log *K*_ow_ values were obtained from <https://www.swissadme.ch/> (average values).

Chemaxon-predicted *p*K_a_ values were obtained primarily from the HMDB website (<https://www.hmdb.ca/>), except where otherwise noted: * values from DrugBank (<https://go.drugbank.com/>), and ** values from ChemicalBook (<https://www.chemicalbook.com/>).

Chemical structures were obtained from <https://www.chemspider.com/>.

**Table S2.** CAS numbers and suppliers of the compounds used in the study.

| **Compound** | **CAS Number** | **Supplier (Brand)** |
| --- | --- | --- |
| Ethyl sulphate | 546-74-7 | Merck (Cerilliant) |
| *cis*-Resveratrol | 61434-67-1 | LGC Standards (TRC) |
| *trans*-Resveratrol | 501-36-0 | LGC Standards (TRC) |
| Isoxanthohumol | 521-48-2 | LGC Standards (TRC) |
| 8-Prenylnaringenin | 68682-02-0 | LGC Standards (TRC) |
| Cotinine | 486-56-6 | Cayman Chemicals |
| Cotinine *N*-β-D-glucuronide | 139427-57-9 | LGC Standards (TRC) |
| *trans*-3'-Hydroxycotinine | 34834-67-8 | Merck (Cerilliant) |
| Anabasine | 53912-89-3 | Merck (Cerilliant) |
| Caffeine | 58-08-2 | LGC Standards (TRC) |
| Paraxanthine | 611-59-6 | Merck (Cerilliant) |
| Sucralose | 56038-13-2 | LGC Standards (TRC) |
| Acesulfame Potassium | 55589-62-3 | LGC Standards (TRC) |
| Saccharin | 81-07-2 | LGC Standards (Dr. Ehrenstorfer) |
| Sodium Cyclamate | 139-05-9 | LGC Standards (Dr. Ehrenstorfer) |
| Enterodiol | 77756-22-0 | Merck (Sigma-Aldrich) |
| Enterolactone | 78473-71-9 | Merck (Sigma-Aldrich) |
| Proline betaine hydrochloride | 4136-37-2 | LGC Standards (TRC) |
| Naringenin | 67604-48-2 | LGC Standards (TRC) |
| Hesperetin | 69097-99-0 | LGC Standards (TRC) |
| Apigenin | 520-36-5 | LGC Standards (TRC) |
| Luteolin | 491-70-3 | LGC Standards (TRC) |
| Sulforaphane *N*-Acetyl cysteine | 334829-66-2 | LGC Standards (TRC) |
| *S*-Methyl-L-cysteine sulfoxide | 6853-87-8 | LGC Standards (TRC) |
| Daidzein | 486-66-8 | LGC Standards (TRC) |
| Equol | 94105-90-5 | LGC Standards (TRC) |
| Genistein | 446-72-0 | LGC Standards (TRC) |
| *O*-desmethylangolensin | 21255-69-6 | LGC Standards (TRC) |
| 3,5-Dihydroxy-4-methoxybenzoic acid | 4319-02-2 | LGC Standards (TRC) |
| 3,5-Dihydroxyphenylpropionoic acid | 26539-01-5 | LGC Standards (TRC) |
| Secoisolariciresinol | 75365-01-4 | LGC Standards (TRC) |
| Tyrosol | 501-94-0 | LGC Standards (TRC) |
| Hydroxytyrosol | 10597-60-1 | LGC Standards (TRC) |
| L-Anserine | 584-85-0 | LGC Standards (TRC) |
| 3-Carboxy-4-methyl-5-propyl-2-furanpropanoic acid | 86879-39-2 | LGC Standards (TRC) |
| Riboflavin | 83-88-5 | Merck (Supelco) |
| 8-Hydroxymethylriboflavin | 52134-62-0 | LGC Standards (TRC) |
| Nicotinamide | 79-83-4 | Supelco |
| Pantothenic acid | 82-82-6 | Merck (Sigma-Aldrich) |
| 4-Pyridoxic acid | 82-82-6 | Merck (Sigma-Aldrich) |
| 2,5,7,8-Tetramethyl-2(2′-carboxyethyl)-6-hydroxychroman | 4072-32-6 | Cayman Chemicals |
| 2,7,8-Trimethyl-2-(beta-carboxyethyl)-6-hydroxychroman | 178167-75-4 | LGC Standards (TRC) |
| Metformin hydrochloride | 1115-70-4 | Merck (Cerilliant) |
| Ethyl sulphate-*d*_5_ | 1329611-05-3 | Merck (Cerilliant) |
| *trans*-Resveratrol-^13^C_6_ | 1185247-70-4 | LGC Standards (TRC) |
| Cotinine-*d*_3_ | 110952-70-0 | LGC Standards (TRC |
| Cotinine *N*-β-D-glucuronide-*d*_3_ | – | LGC Standards (TRC) |
| *trans*-3'-Hydroxycotinine-*d*_3_ | 159956-78-2 | Merck (Cerilliant) |
| Anabasine-*d*_4_ | – | Merck (Cerilliant) |
| Caffeine-^13^C_3_ | 78072-66-9 | LGC Standards (TRC) |
| Sucralose-*d*_6_ | 1459161-55-7 | LGC Standards (TRC) |
| Acesulfame-*d*_4_ Potassium | 1623054-53-4 | LGC Standards (TRC) |
| Saccharin-*d*_4_ | 1189466-17-8 | LGC Standards (TRC) |
| Sodium Cyclamate-*d*_11_ | – | LGC Standards (CDN Isotopes) |
| Enterodiol-^13^C_3_ | 918502-74-6 | LGC Standards (TRC) |
| Enterolactone-^13^C_3_ | 918502-72-4 | LGC Standards (TRC) |
| Apigenin-*d*_5_ | 263711-74-6 | LGC Standards (TRC) |
| *S*-Methyl-L-cysteine sulfoxide-*d*_3_ | – | LGC Standards (TRC |
| Daidzein-*d*_6_ | 291759-05-2 | LGC Standards (TRC) |
| Equol-*d*_4_ | 1216469-13-4 | LGC Standards (TRC) |
| Genistein-*d*_4_ | 187960-08-3 | LGC Standards (TRC) |
| Tyrosol-*d*_4_ | – | LGC Standards (TRC) |
| Hydroxytyrosol-*d*_4_ | 1330260-89-3 | LGC Standards (TRC) |
| 3-Carboxy-4-methyl-5-propyl-2-furanpropanoic acid-*d*_3_ | – | LGC Standards (TRC) |
| Riboflavin-^13^C_4_,^15^N_2_ | 1217461-14-7 | Merck (Supelco) |
| *N*-methyl-4-pyridone-5-carboxamide-*d*_3_ | 1207384-47-1 | LGC Standards (TRC) |
| 4-Pyridoxic acid-*d*_3_ | 1435934-03-4 | LGC Standards (TRC) |
| 2,7,8-Trimethyl-2-(beta-carboxyethyl)-6-hydroxychroman-*d*_5_ | – | LGC Standards (TRC) |
| Metformin-*d*_6_ Hydrochloride | 1185166-01-1 | LGC Standards (TRC) |

TRC = Toronto Research Chemicals

**Table S3.** Stability of target biomarkers in wastewater during storage at –18 °C (freeze–thaw stability).

| **Compound** | **Residual after 30 days / %** | **Residual after 95 days / %** |
| --- | --- | --- |
| Ethyl sulfate | 119 ± 0 | 122 ± 5 |
| *cis*-Resveratrol | 78 ± 15 | 87 ± 12 |
| *trans*-Resveratrol | 69 ± 7 | 69 ± 11 |
| Isoxanthohumol | 120 ± 12 | 31 ± 7 |
| 8-Prenylnaringenin | 116 ± 8 | 20 ± 20 |
| Cotinine | 114 ± 0 | 112 ± 5 |
| Cotinine glucuronide | 91 ± 1 | 86 ± 5 |
| *trans*-3'-Hydroxycotinine | 107 ± 1 | 104 ± 4 |
| Anabasine | 100 ± 6 | 99 ± 5 |
| Caffeine | 107 ± 1 | 108 ± 5 |
| Paraxanthine | 105 ± 1 | 109 ± 4 |
| Sucralose | 107 ± 3 | 106 ± 3 |
| Acesulfame | 108 ± 3 | 112 ± 3 |
| Saccharin | 111 ± 1 | 109 ± 6 |
| Cyclamate | 113 ± 3 | 111 ± 5 |
| Enterodiol | 117 ± 1 | 106 ± 4 |
| Enterolactone | 112 ± 1 | 107 ± 5 |
| Proline betaine | 111 ± 0 | 95 ± 5 |
| Naringenin | 45 ± 14 | 14 ± 5 |
| Hesperetin | 43 ± 12 | 15 ± 10 |
| Apigenin | 29 ± 1 | 22 ± 7 |
| Luteolin | 60 ± 5 | 17 ± 7 |
| Sulforaphane *N*-Acetyl cysteine | 56 ± 47 | 34 ± 4 |
| *S*-Methyl-L-cysteine sulfoxide | 85 ± 5 | 75 ± 5 |
| Daidzein | 123 ± 3 | 72 ± 5 |
| Equol | 113 ± 4 | 116 ± 5 |
| Genistein | 87 ± 3 | 70 ± 3 |
| *O*-desmethylangolensin | 76 ± 14 | 54 ± 5 |
| 3,5-Dihydroxy-4-methoxybenzoic acid | 13 ± 41 | 51 ± 1 |
| 3,5-Dihydroxyphenylpropionoic acid | 89 ± 34 | 48 ± 3 |
| Secoisolariciresinol | 94 ± 18 | 102 ± 2 |
| Tyrosol | 89 ± 4 | 65 ± 6 |
| Hydroxytyrosol | 3 ± 34 | 0 ± 0 |
| Anserine | 120 ± 8 | 74 ± 1 |
| 3-Carboxy-4-methyl-5-propyl-2-furanpropanoic acid | 108 ± 2 | 92 ± 6 |
| Riboflavin | 88 ± 1 | 66 ± 4 |
| 8-Hydroxymethylriboflavin | 99 ± 1 | 65 ± 2 |
| Nicotinamide | 20 ± 23 | 22 ± 4 |
| Pantothenic acid | 121 ± 5 | 126 ± 3 |
| 4-Pyridoxic acid | 105 ± 1 | 100 ± 5 |
| 2,5,7,8-Tetramethyl-2(2′-carboxyethyl)-6-hydroxychroman | 103 ± 2 | 115 ± 43 |
| 2,7,8-Trimethyl-2-(beta-carboxyethyl)-6-hydroxychroman | 76 ± 6 | 60 ± 12 |
| Metformin | 101 ± 1 | 108 ± 6 |

**Table S4.** Concentrations of target compounds (µg L⁻¹) in raw wastewater samples collected from WWTPs in Croatian cities on specified dates.

| **Compound abbreviation** | **Karlovac** | **Čakovec** | | **Zagreb** | | **Bjelovar** |
| --- | --- | --- | --- | --- | --- | --- |
|  | **22/01/2025** | **22/01/2025** | **12/11/2025** | **03/11/2025** | **05/11/2025** | **12/11/2025** |
| **EtS** | 6.4 | 13 | 8.3 | 8.4 | 7.0 | 6.0 |
| **c-RES** | 0.040 | 0.035 | 0.12 | 0.097 | 0.053 | 0.11 |
| **t-RES** | 0.028 | 0.059 | 0.054 | 0.032 | 0.049 | 0.037 |
| **IXH** | 0.028 | 0.10 | 0.11 | 0.29 | 0.35 | 0.050 |
| **PRE** | 0.012 | 0.010 | 0.027 | 0.028 | 0.022 | 0.012 |
| **COT** | 1.1 | 2.7 | 4.9 | 2.8 | 3.3 | 3.7 |
| **COT-Glu** | 1.9 | 3.6 | 1.9 | 1.5 | 2.6 | 1.3 |
| **COT-OH** | 3.2 | 7.5 | 8.6 | 5.9 | 6.7 | 7.0 |
| **ANB** | 0.40 | 0.31 | 0.70 | 0.72 | 0.70 | 0.65 |
| **CAF** | 41 | 81 | 61 | 56 | 64 | 53 |
| **PAR** | 13 | 29 | 38 | 28 | 34 | 31 |
| **SUC** | 13 | 32 | 27 | 13 | 15 | 25 |
| **ACE** | 9.8 | 26 | 27 | 21 | 24 | 30 |
| **SAC** | 7.3 | 18 | 15 | 9.7 | 12 | 25 |
| **CYC** | 19 | 38 | 30 | 30 | 33 | 90 |
| **EDL** | 0.31 | 0.84 | 0.82 | 0.54 | 0.49 | 0.45 |
| **ELC** | 0.95 | 2.5 | 3.0 | 1.5 | 1.3 | 1.5 |
| **PB** | 4.2 | 24 | 7.7 | 11 | 22 | 5.5 |
| **NAR** | 0.53 | 1.5 | 1.2 | 1.2 | 2.5 | 1.1 |
| **HES** | 0.84 | 2.1 | 1.2 | 1.8 | 3 | 1.8 |
| **API** | 0.12 | 0.38 | 1.2 | 1.3 | 2.7 | 1.2 |
| **LUT** | 0.057 | 0.13 | 0.089 | 0.068 | 0.068 | 0.053 |
| **SNC*** | 0.10 | 0.19 | 0.12 | 0.15 | 0.16 | 0.13 |
| **MCS** | 2.4 | 9.5 | 2.2 | 4.1 | 11 | 1.6 |
| **DAI** | 0.33 | 4.4 | 5.1 | 0.40 | 0.88 | 0.48 |
| **EQ** | 0.031 | 2.5 | 1.3 | 0.051 | 0.041 | 0.10 |
| **GEN** | 0.11 | 1.4 | 1.6 | 0.19 | 0.53 | 0.15 |
| **DMA** | 0.038 | 0.53 | 0.19 | 0.05 | 0.06 | 0.071 |
| **DMBA** | 0.62 | 0.84 | 1.9 | 1.4 | 1.3 | 4.5 |
| **DHPPA** | 0.91 | 9 | 4.4 | 5.1 | 10.4 | 2.9 |
| **SEC** | 0.26 | 0.36 | 0.33 | 0.33 | 0.37 | 1.7 |
| **TYR** | 1.4 | 6.6 | 5.2 | 5.0 | 4.6 | 6.1 |
| **TYR-OH** | 0.17 | 0.81 | 0.65 | 0.81 | 0.51 | 0.47 |
| **ANS** | 2.5 | 33 | 62 | 8.1 | 20 | 11 |
| **CMPF** | 0.12 | 0.33 | 0.21 | 0.12 | 0.13 | 0.12 |
| **B2** | 3.2 | 8.8 | 7.9 | 3.7 | 4.5 | 3.7 |
| **B2-OH** | 0.018 | 0.044 | 0.15 | 0.095 | 0.11 | 0.15 |
| **NAMD** | 1.0 | 1.0 | 1.5 | 11 | 0.68 | 1.2 |
| **B5** | 5.3 | 16 | 50 | 122 | 46 | 66 |
| **4-PA** | 0.076 | 0.095 | 7.1 | 4.2 | 5.0 | 5.7 |
| **α-CEHC** | N/A | N/A | 3.5 | 2.4 | 2.2 | 2.2 |
| **γ-CEHC** | 1.7 | 5.5 | 5.3 | 2.1 | 2.8 | 1.9 |
| **MET** | N/A | N/A | 83 | 77 | 76 | 87 |

N/A − concentration not determined due to their later inclusion in the analytical method

* Results should be interpreted as semi-quantitative

**Fig S1.** Locations of wastewater sampling.

**NEGATIVE POLARITY**

**POSITIVE POLARITY**

**Fig. S2.** MRM chromatograms of the standard mixture of the target compounds (10 ng mL^-1^) obtained by LC–MS/MS using an HSS T3 column (100 × 2.1 mm, 1.8 µm). Retention times and quantification transitions for each analyte are shown.

**Fig. S3.** Filtration loss of target analytes after filtering spiked spring water (spiking level 10 µg L^-1^; *n*=4) through different syringe filter materials: regenerated cellulose (RC), glass microfiber (GMF), polyvinylidene fluoride (PVDF), hydrophilic polytetrafluoroethylene (wwPTFE), polyethersulphone (PES), polytetrafluoroethylene (PTFE), and polypropylene (PP).
